# Efficacy Evaluation of Bevacizumab Combined with Capecitabine in the Treatment of HER2-Negative Metastatic Breast Cancer: A Meta-Analysis

**DOI:** 10.1155/2023/8740221

**Published:** 2023-02-08

**Authors:** Yiyi Hu, Peizhen Chen, Feng Xiang

**Affiliations:** Department of Pharmacy, The Second Affiliated Hospital of Wenzhou Medical University, Wenzhou 325027, Zhejiang, China

## Abstract

**Objective:**

This study aims to evaluate the efficacy of bevacizumab combined with capecitabine in treating HER2-negative metastatic breast cancer through meta-analysis.

**Methods:**

We searched literature from databases, including PubMed, Web of Science, Wiley Online Library, Ovid, CNKI, and Wanfang databases, for randomized controlled trials (RCTs) of bevacizumab combined with capecitabine (experimental group) and other treatments (control group) for HER2-negative metastatic breast cancer. Retrieved articles were published from the establishment of the database to August 9, 2022. The main outcome indicators were disease progression rate (RDP), disease progression-free survival (PFS), 1-year survival rate (OSR), the occurrence of serious adverse events (SAEs), and objective remission rate (ORR). The risk of bias was assessed according to the Cochrane systematic evaluation tool. Then, the meta-analysis was carried out using Stata16.0 software, and subgroup analysis was carried out based on various intervention methods in the control group.

**Results:**

8 RCTs were finally included in this study, including 2470 patients with HER2-negative metastatic breast cancer. The results of meta-analysis showed that bevacizumab combined with capecitabine had no significant advantage over the control group in terms of RDP, but the results of subgroup analysis were consistent and significant (subgroup 1 (bevacizumab or chemotherapy): DR = −0.03, 95% CI (−0.14, 0.09), *P* = 0.01; subgroup 2 (bevacizumab plus paclitaxel therapy): DR = −0.03, 95% CI (−0.14, 0.09), *P* = 0.03). Furthermore, there was no statistical difference in terms of PFS of the experimental group (MD = 9.24, 95% CI (7.88, 32.67), *P* = 0.05). However, the subgroup analysis showed that the combination of bevacizumab and capecitabine demonstrated a more significant significance than bevacizumab or chemotherapy alone (subgroup 1: MD = 10.11, 95% CI (7.88, 12.34), *P* = 0.00). Compared with the control group, the experimental group had significant differences in OSR (DR = 0.07, 95% CI (−0.01, 0.15), *P* = 0.00) and ORR (DR = 0.07, 95% CI (−0.01, 0.15), *P* = 0.00). In terms of safety, the incidence of serious adverse events in the experimental group did not show a statistically significant difference (MD = 0.01, 95% CI (−0.21, 0.19), *P* = 0.82). When subgroup analyses were performed, the bevacizumab plus capecitabine regimen was associated with an increased incidence of serious adverse events compared with the drug alone (subgroup 1: MD = 0.02, 95% CI (−0.16, 0.20), *P* = 0.00) but a reduction in serious adverse events compared with the bevacizumab plus paclitaxel regimen (subgroup 2: DR = −0.01, 95% CI (−0.21, 0.19), *P* = 0.00).

**Conclusion:**

The combination therapy of bevacizumab and capecitabine can significantly improve the RDP and OSR of patients compared with the control group. The PFS and ORR of the experimental group are significantly higher than those of bevacizumab or chemotherapy alone. Still, no statistical difference was observed for these outcome indicators between two combined treatments of bevacizumab with capecitabine or paclitaxel. Although this combined treatment scheme may increase the incidence of serious adverse events compared with that of bevacizumab or chemotherapy alone, the incidence of adverse events was decreased compared with bevacizumab combined with paclitaxel. Therefore, the chemotherapy regimen for HER2-negative metastatic breast cancer in clinical practice can be selected according to the actual situation of the patients.

## 1. Introduction

Breast cancer is one of the most common cancer malignancies in women. About 5–10% of breast cancer patients have metastatic disease, while 20–40% of early breast cancer patients eventually develop metastatic breast cancer [1]. Advanced tumors, especially HER2-negative metastatic breast cancer, are generally considered to be incurable despite the continuous development of therapeutic drugs [2–4]. The prognosis of metastatic breast cancer is also relatively poor. The median survival period of patients is only 2-3 years, and the 5-year survival rate is 23–26%. Therefore, metastatic breast cancer remains a major medical challenge [4]. For HER2-negative local recurrent or metastatic breast cancer, the first-line treatment options in Europe include single-agent chemotherapy and bevacizumab combined with chemotherapy drugs (paclitaxel or capecitabine). As a monoclonal antibody targeting tumor angiogenesis, bevacizumab has been proven to improve the prognosis of many metastatic cancers by inhibiting vascular endothelial growth factor (VEGF) [5, 6]. It has also been proved to be effective in the treatment of metastatic breast cancer. For example, the combination of taxane therapy and phase III clinical trial of HER2-negative local recurrence or metastatic breast cancer significantly improves the disease progression-free survival (PFS) and objective remission rate (ORR) compared with taxane therapy alone [7]. Compared with capecitabine and placebo, the combination of first-line chemotherapy drugs capecitabine and bevacizumab has also significantly improved PFS and ORR [8]. However, a previous study has suggested that bevacizumab has no benefit in the survival time of patients with HER2-negative metastatic breast cancer undergoing combined chemotherapy [9]. Therefore, there is no consensus on the efficacy of bevacizumab combined with capecitabine compared with chemotherapy alone or combined with other chemotherapy drugs. Therefore, we used the method of meta-analysis to evaluate the efficacy of bevacizumab combined with capecitabine in treating HER2-negative metastatic breast cancer. This study may provide a theoretical basis for selecting a drug regimen for clinical patients with HER2-negative metastatic breast cancer.

## 2. Materials and Methods

### 2.1. Data Retrieval

We searched literature from the English databases PubMed, Web of Science, Wiley Online Library, and Ovid, as well as the Chinese databases CNKI and Wanfang. The retrieved articles were about the treatment of HER2-negative metastatic breast cancer with bevacizumab and capecitabine and were published before August 9, 2022. The search keywords were “Bevacizumab,” “Capecitabine,” “HER2 negative,” “Breast cancer,” “Metastatic,” etc.

### 2.2. Literature Inclusion and Exclusion Criteria

Literature inclusion criteria were as follows: (1) patients were diagnosed with HER2-negative metastatic breast cancer; (2) the research type of the article was a randomized controlled study (RCTs); (3) the intervention mode of the experimental group was bevacizumab combined with capecitabine, and the control group was bevacizumab or chemotherapy drugs alone or in combination; and (4) the main outcome measures included disease progression rate (RDP), disease progression-free survival (PFS), 1-year survival rate (OSR), objective remission rate (ORR), and serious adverse events (SAEs). Exclusion criteria were as follows: (1) case reports and literature reviews; (2) data of main outcome indicators could not be obtained; and (3) patients received other intervention methods besides bevacizumab and capecitabine during the study period. The two researchers independently screened the literature in strict accordance with the inclusion and exclusion criteria. In case of disagreement, they joined the third researcher to discuss and decide.

### 2.3. Data Extraction

Two researchers, respectively, extracted the basic data of the included literature. The basic information of the extracted literature included (1) the first author, the year of publication, the type of study, the number of patients in each group, the age of patients in each group, the intervention plan of patients in each group, and the inclusion outcome indicators. (2) Main outcome measures included the following: disease progression rate (RDP), disease progression-free survival (PFS), 1-year survival rate (OSR), serious adverse events (SAEs), and objective remission rate (ORR). Data that did not conform to the input format were converted based on the calculation method published by Luo et al. [10, 11] (see Table 1 for details).

### 2.4. Risk Offset Assessment of Included Documents

The guidelines published in Cochrane Handbook [12, 13] were used to evaluate the quality of the literature: (1) selection bias; (2) group hiding; (3) blind method for both doctors and patients; (4) blinded method of outcome evaluation; (5) completeness of the report results; (6) publication bias; and (7) other indicators in the paper include low risk, high risk, and unclear risk. As shown in Table 2, ≤1 of the 7 items was assessed as “high risk” or “unclear,” and ≤2 items were assessed as “high risk” or “unclear.” More than 2 items are low-quality articles.

### 2.5. Statistical Analysis

Meta combined effect value analysis was performed with Stata software 16.0. The heterogeneity among the included studies was judged by the *Q* test and *I*^2^ test. In the *Q* test, it was considered that the studies were homogeneous, and the fixed effect model was adopted when *P* > 0.1 and *I*^2^ < 50%. On the contrary, it was considered that the studies were heterogeneous, and the random effect model was adopted. Subgroup analysis was conducted to explore the source of heterogeneity. RDP, OSR, SAE, and ORR were dichotomous variables in outcome indicators. Thus, the difference ratio (DR) was used as the combined effect value. PFS is a continuous variable, and mean difference (MD) was used as the combined effect value. The difference was statistically significant if *P* < 0.05.

## 3. Results

### 3.1. Inclusion of Literature and Assessment of Risk of Bias

**Figure 1:**
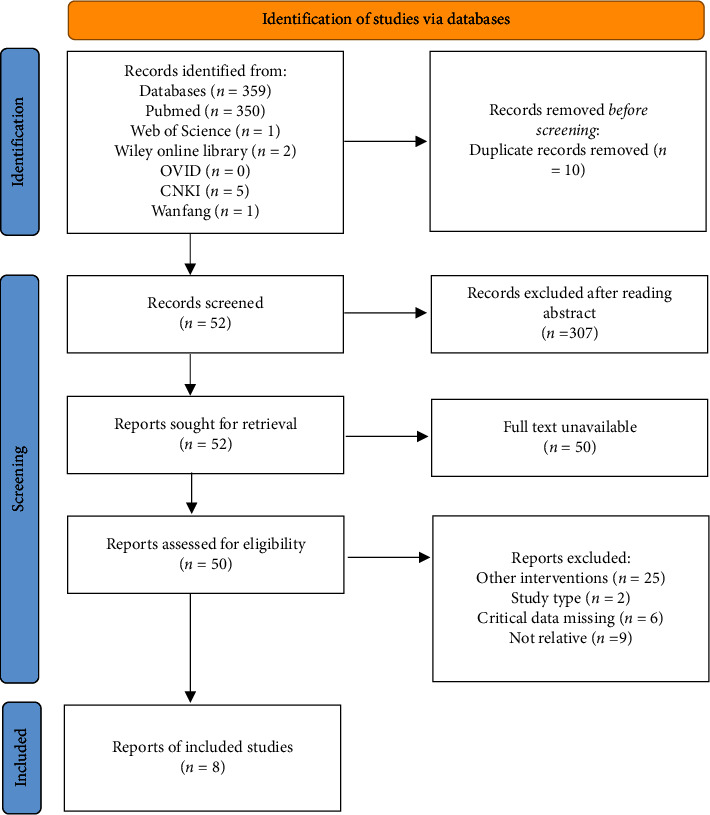
Flowchart of inclusion process.

The preliminary literature search in the database found that 353 English and 6 Chinese literature met the inclusion conditions. After de-duplication of the screened literature by software, 349 articles remained. After reading the titles and abstracts of these articles, 307 articles were removed. Of the remaining 52 articles, 50 could be obtained and entered into the full-text screening. Finally, 8 articles were included in the study with 2470 patients with HER2-negative metastatic breast cancer (Figure 1). After the risk of bias assessment of the literature, it was found that only one of the 5 articles was “high risk” or “unclear,” which was designated as high-quality literature. Only two of the three articles were rated as “high risk” or “unclear” and were rated as moderate quality.

### 3.2. Disease Progression Rate (RDP)

**Figure 2:**
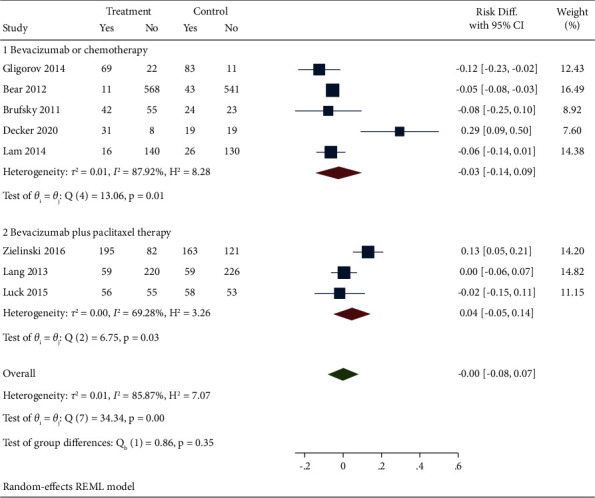
Forest diagram and subgroup analysis of bevacizumab combined with capecitabine for RDP results.

Eight studies reported the efficacy of bevacizumab combined with capecitabine compared with the RDP results of bevacizumab and chemotherapy drugs used alone or in combination. According to the meta combined effect value analysis, there was heterogeneity among the 8 included studies (*I*^2^ = 85.87%). A random effect model was adopted, and a subgroup analysis was conducted. The results showed that the combination of bevacizumab and capecitabine had no significant advantage in RDP compared with the control group (DR = −0.00, 95% CI (−0.08, 0.07), *P*=0.35). In subgroup analysis, different treatment methods of the control group (1: single bevacizumab or chemotherapy drug treatment; 2: bevacizumab combined with paclitaxel treatment) were compared. The results indicated that subcombinations were found to have good consistency (subgroup 1: DR = −0.03, 95% CI (−0.14, 0.09), *P*=0.01; subgroup 2: DR = −0.03, 95% CI (−0.14, 0.09), *P*=0.03), and both had significant differences. After analyzing the heterogeneity of the included literature, it was found that the study of Decker et al. [19] was the main source of heterogeneity, as shown in Figure 2.

### 3.3. Disease Progression-Free Survival (PFS)

**Figure 3:**
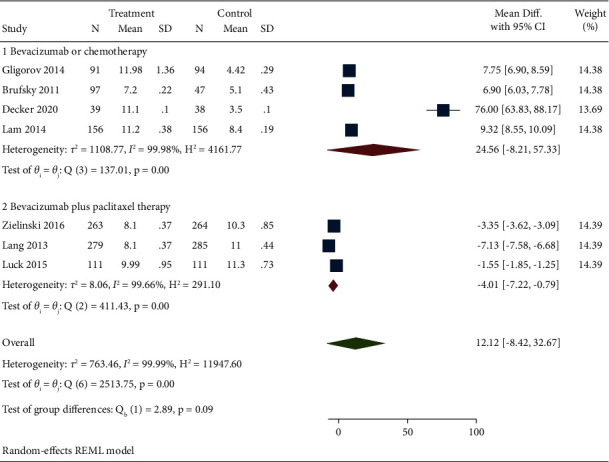
Forest chart and subgroup analysis of bevacizumab combined with capecitabine for PFS results.

Seven studies reported the PFS results of bevacizumab combined with capecitabine compared with bevacizumab and chemotherapy drugs alone or in combination. Meta-analysis combined value analysis found a large heterogeneity between the studies (*I*^2^ = 91.25%). The random effect model was used, and subgroup analysis was conducted. The results demonstrated no statistical difference in PFS compared between the experimental and the control group (MD = 9.24, 95% CI (7.88, 32.67), *P* = 0.05). However, when performing subgroup analysis according to different treatment methods of patients in the control group, it was found that bevacizumab plus capecitabine treatment had better significance compared with bevacizumab or chemotherapy alone (subgroup 1: MD = 10.11, 95% CI (7.88, 12.34), *P* = 0.00), but there was no difference in PFS compared with bevacizumab plus paclitaxel regimen (subgroup 2: DR = 7.72, 95% CI (6.9, 8.55), *P* = 0.27). After analyzing the source of heterogeneity, it is found that it is consistent with the source of heterogeneity in Section 3.2, from the study of Decker et al. [19] (see Figure 3 for details).

### 3.4. One-Year Survival Rate (OSR)

**Figure 4:**
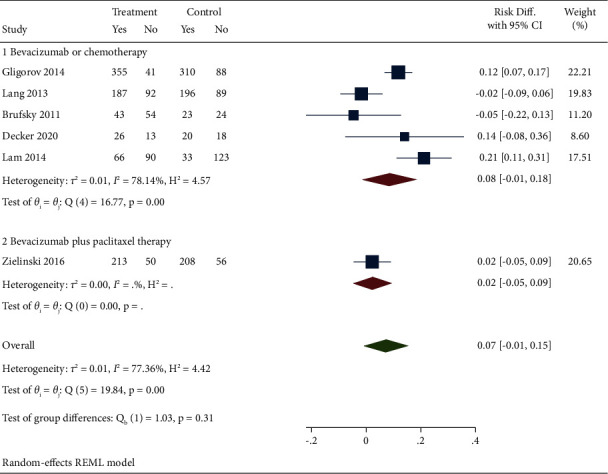
Forest chart and subgroup analysis of bevacizumab combined with capecitabine treatment for ORS results.

Five studies reported the OSR results of bevacizumab combined with capecitabine compared with bevacizumab and chemotherapy drugs alone or in combination. Meta-analysis found heterogeneity among the studies (*I*^2^ = 77.36%). A random effect model was used, and a subgroup analysis was conducted. The results showed that it was significant in OSR (DR = 0.07, 95% CI (−0.01, 0.15), *P*=0.00) between the experimental and the control groups. In subgroup analysis, it was also found that the bevacizumab plus capecitabine regimen also had better significance compared with bevacizumab or chemotherapy alone (subgroup 1: RD = 0.08, 95% CI (−0.01, 0.18), *P*=0.00). There was only one kind of data in the bevacizumab plus paclitaxel group, so the effect value could not be combined. After analyzing the heterogeneity of the included studies in this part, it was found that it may be due to the adjustment of the treatment plan after the patients developed serious complications and the differences in the countries and medical units where the patients were located, as shown in Figure 4.

### 3.5. Objective Response Rate (ORR)

**Figure 5:**
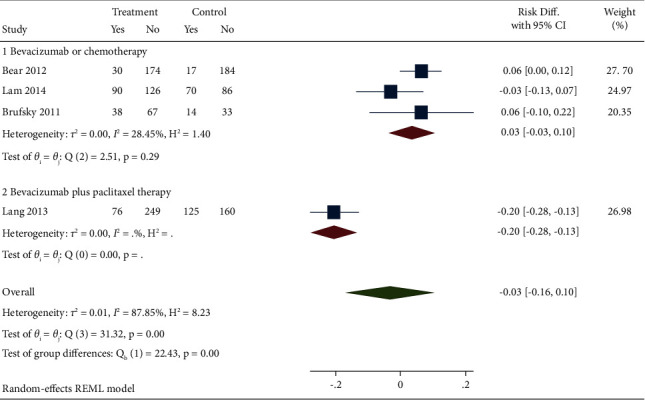
Forest chart and subgroup analysis of bevacizumab combined with capecitabine for ORR results.

Five studies reported the ORR results of bevacizumab combined with capecitabine compared with bevacizumab and chemotherapy drugs alone or in combination. Meta-analysis combined effect value analysis found heterogeneity among the studies (*I*^2^ = 87.85%). A random effect model was used, and a subgroup analysis was conducted. The results showed that ORR was significantly different between groups (DR = 0.07, 95% CI (−0.01, 0.15), *P* = 0.00). However, in subgroup analysis, it was also found that there was no statistical difference between bevacizumab plus capecitabine regimen and bevacizumab or chemotherapy alone (subgroup 1: RD = 0.03, 95% CI (−0.03, 0.10), *P* = 0.29). Since only one article was about bevacizumab plus paclitaxel, the effect value could not be combined, as shown in Figure 5.

### 3.6. Serious Adverse Events (SAEs)

**Figure 6:**
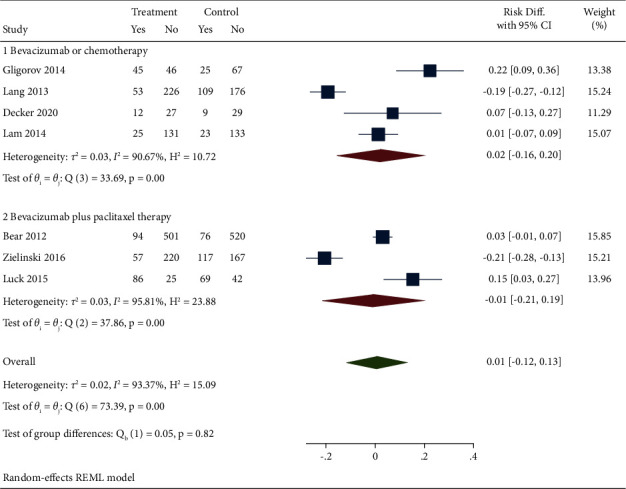
Forest chart and subgroup analysis of bevacizumab combined with capecitabine for SAE results.

Seven studies reported the SAE results of bevacizumab combined with capecitabine compared with bevacizumab and chemotherapy drugs alone or in combination. Meta-analysis combined effect value analysis found heterogeneity among studies (*I*^2^ = 93.37%). A random effect model was used, and a subgroup analysis was conducted. The results showed no statistical difference in SAE between the experimental group and the control group (MD = 0.01, 95% CI (−0.21, 0.19), *P*=0.82). However, when performing subgroup analysis according to different treatment methods of patients in the control group, bevacizumab plus capecitabine regimen showed an increased incidence of serious adverse events compared with bevacizumab or chemotherapy alone (subgroup 1: MD = 0.02, 95% CI (−0.16, 0.20), *P*=0.00). However, the incidence of serious adverse events was relatively small, and the difference was statistically significant (subgroup 2: DR = −0.01, 95% CI (−0.21, 0.19), *P*=0.00) compared to bevacizumab combined with paclitaxel. After analyzing the heterogeneity of the included studies in this part, it was found that it may be due to the adjustment of the treatment plan after the patient developed serious complications and the difference in the evaluation of adverse reactions by medical institutions in different countries, as shown in Figure 6.

## 4. Discussion

The treatment of breast cancer is still a complex problem. The current guidelines indicate that endocrine therapy is the preferred treatment for most HER2-positive and HER2-negative metastatic patients [21]. In fact, 43% of clinical patients mainly receive chemotherapy [22]. When capecitabine was combined with bevacizumab to treat HER2-negative metastatic breast cancer, researchers found that the objective remission rate of patients increased, but the disease progression-free survival period did not change [23]. Other studies have reported that bevacizumab combined with paclitaxel or capecitabine has good and controllable safety [23, 24]. However, the superiority of bevacizumab combined with capecitabine has not been relatively unified. Therefore, our study selected the relevant literature on bevacizumab combined with capecitabine for the combined effect evaluation.

In our study, the combination therapy of bevacizumab and capecitabine could significantly improve the disease progression rate and one-year survival rate of patients compared with the control group. For the disease progression-free survival rate and objective remission rate, the combination therapy of bevacizumab and capecitabine in the experimental group has a significant improvement compared with bevacizumab or chemotherapeutic drugs alone. Still, there is no statistical difference compared with the combination therapy of bevacizumab and paclitaxel. In terms of drug safety, although the treatment regimen in the experimental group increased the incidence of serious adverse events compared with bevacizumab or chemotherapy alone, the incidence of adverse events decreased compared with bevacizumab plus paclitaxel treatment regimen. Therefore, we suggested that the treatment effect of bevacizumab combined with capecitabine was significantly improved compared with that of bevacizumab or chemotherapy alone, but the incidence of serious adverse events was increased simultaneously. When bevacizumab combined with capecitabine was compared with that of bevacizumab combined with paclitaxel, the incidence of serious adverse events in patients also decreased.

This meta-analysis conducted a clear and comprehensive search on the treatment of HER2-negative metastatic breast cancer with bevacizumab combined with capecitabine and performed a subgroup analysis on different treatment schemes. However, some heterogeneity was not analyzed. For example, the current treatment scheme is relatively new, and the published literature is relatively small, resulting in a small number of included samples. There was no completely unified standard for the dosage of each study, and the studies were from different parts of the world, with ethnic and regional differences. Therefore, the evaluation of bevacizumab combined with capecitabine needs to be included in more comprehensive literature for more in-depth research.

To sum up, it was found that the treatment effect of bevacizumab combined with capecitabine was significantly improved compared with that of bevacizumab or chemotherapy alone, but the incidence of serious adverse events was increased at the same time. Compared with bevacizumab combined with paclitaxel, the treatment effect was improved, and the incidence of serious adverse events was reduced to a certain extent. In the clinical use of drugs, the appropriate drug treatment scheme can be selected according to the actual situation of the patients.

## Figures and Tables

**Table 1:** Basic information of patients included in the study.

Authors	Study type	Sample size		Age		Therapeutic regimen		Subgroup	Outcome indicators
		E	C	E	C	E	C		
Gligorov et al. [14]	RCT	91	94	49 (24–80)	54 (24–77)	Bevacizumab + capecitabine	Bevacizumab	1	①②③④
Bear et al. [15]	RCT	204	201	39.6 (/–/)	40.3 (/–/)	Bevacizumab + capecitabine-docetaxel	Capecitabine-docetaxel	1	①③⑤
Zielinski et al. [16]	RCT	277	284	≥18	≥18	Bevacizumab + capecitabine	Bevacizumab + paclitaxel	2	①②③④
Lang et al. [17]	RCT	279	285	59 (48–65)	59 (49–65)	Bevacizumab + capecitabine	Bevacizumab + paclitaxel	2	①②③④⑤
Brufsky et al. [18]	RCT	97	47	57 (31–78)	50 (23–90)	Bevacizumab + capecitabine	Capecitabine + placebo	1	①②③⑤
Decker et al. [19]	RCT	39	38	64.4 (47–83.6)	65.9 (49.8–86)	Bevacizumab + capecitabine	Everolimus + exemestane	1	①②③④
Lam et al. [8]	RCT	156	156	56 (32–76)	56 (34–74)	Bevacizumab + capecitabine-docetaxel	Capecitabine-docetaxel	1	①②③④⑤
Luck et al. [20]	RCT	111	111	56.2 (31–78)	57 (33–80)	Bevacizumab + capecitabine-taxanes	Bevacizumab-taxanes	2	①②④

E, experimental group; C, control group; RCT, randomized controlled trial; /, no data; subgroup 1, control group (non-bevacizumab and chemotherapy drug combination); subgroup 2, control group (bevacizumab and chemotherapy drug combination). Outcome measures: ① disease progression rate; ② disease progression-free survival; ③ 1-year survival rate; ④ incidence of adverse events; ⑤ objective remission rate.

**Table 2:** Risk of bias assessment.

Authors	Random sequence generation	Allocation concealment	Blinding of participants and personnel	Blinding of outcome assessment	Incomplete outcome data	Selective reporting	Other bias	Quality of the literature
Gligorov et al. [14]	Low risk	Low risk	High risk	Low risk	Low risk	Unclear risk	Low risk	Medium
Bear et al. [15]	Low risk	Low risk	Unclear risk	Low risk	High risk	Low risk	Low risk	Medium
Zielinski et al. [16]	Low risk	Low risk	High risk	Low risk	Low risk	Unclear risk	Low risk	High
Lang et al. [17]	Low risk	Low risk	High risk	Low risk	Low risk	Low risk	Low risk	High
Brufsky et al. [18]	Low risk	Low risk	Low risk	Low risk	Low risk	Low risk	Low risk	High
Decker et al. [19]	Low risk	Low risk	High risk	Low risk	Low risk	High risk	Low risk	Medium
Lam et al. [8]	Low risk	Low risk	High risk	Low risk	Low risk	Low risk	Low risk	Medium
Luck et al. [20]	Low risk	Low risk	Low risk	Low risk	High risk	Low risk	Low risk	High

## Data Availability

The labeled dataset used to support the findings of this study is available from the corresponding author upon request.
